# Infant Death due to Cannabis Ingestion

**DOI:** 10.1002/dta.3904

**Published:** 2025-05-21

**Authors:** Donata Favretto, Antonello Cirnelli, Roberto Pertile, Raffaella Stimamiglio, Clara Cestonaro, Oriana Cuman, Anna Pagliaro, Fabio Mattiazzi, Cristina Basso, Maddalena Galeazzi

**Affiliations:** ^1^ Legal Medicine and Toxicology, Department of Cardiac, Thoracic, Vascular Sciences and Public Health University of Padua Padua Italy; ^2^ Specialist in Legal Medicine Portogruaro Italy; ^3^ Specialist in Legal Medicine Padua Italy; ^4^ Cardiovascular Pathology, Department of Cardiac, Thoracic, Vascular Sciences and Public Health University of Padua Padua Italy

**Keywords:** cannabis, infant death, intoxication, tetrahydrocannabinol

## Abstract

A child died in the emergency room of a local hospital a few hours after ingesting a substance the color of cork and the consistency of earth. At home, a modest amount of resinous substance was found. At the hospital, the child exhibited alterations in walking, balance, and consciousness. Intubation was needed for the onset of dyspnea, so fentanyl and ketamine were administered during the procedure. A sample of blood was also collected for clinical investigation. During the autopsy, cadaveric samples were collected. Autopsy evaluation revealed multiorgan congestion in the brain, lung, liver, and kidney. Histological investigations were inconclusive. A thorough toxicological investigation was undertaken by immunochemical technique, gas chromatography–mass spectrometry (GC–MS), and liquid chromatography–tandem mass spectrometry (UPLC–MS/MS) on samples of blood, bile, urine, organs, gastric content, and head hair. Toxicological analysis detected cannabinoids, ketamine, norketamine, and fentanyl in blood drawn from the emergency room. Cannabinoids were also observed in postmortem central blood, peripheral blood, urine, bile, brain, lung, and liver samples. Hair analysis showed tetrahydrocannabinol, cannabidiol, cannabinol, methadone and metabolites, cocaine and metabolites, ketamine, morphine, 6‐monoacetylmorphine, and fentanyl. Gastric content revealed traces of cannabis products. Acute cannabis intoxication in the context of chronic exposure to numerous drugs has been considered responsible for the death. An increasing number of intoxication cases are being reported worldwide due to the legalization of cannabis. In most cases, these are adults suffering from preexisting conditions, whereas data on younger individuals are still scarce. In this paper, the case of a child who died from acute intoxication due to ingestion of hashish is presented.

## 1 Introduction

Although recreational use of cannabis is illegal in most world countries, this remains the most widely used drug worldwide, with about 190 million consumers [1, 2, 3]. From the cannabis plant, identified as *Cannabis sativa* L., hashish (resin), marijuana (flowers and leaves), or oil (concentrated resin) are obtained [1]. The most intoxicating substance in cannabis is delta‐9‐tetrahydrocannabinol (THC), which is considered the active ingredient [4]. The different parts of the plant, which collectively contains almost 500 different chemical compounds called cannabinoids, contain varying concentrations of THC, which is formed via decarboxylation of tetrahydrocannabinolic acid (THCA) from the application of heat, ranging from less than 10% (leaves) to more than 30% (hashish resin) [1, 5]. Unlike cannabinol (CBN) and cannabidiol (CBD), almost without effects on the central nervous system (CNS) [6], the binding of THC, as a partial agonist to the CB‐1 receptor, exerts its main effects on the CNS [6, 7].

The main effects of THC on the human body certainly depend on the dose, mode of intake, combined intake with other substances, body weight, and interindividual variability [1, 8]. Individuals who normally use cannabis for recreational purposes seek an altered state of consciousness in association with emotional (relaxation, euphoria) or perceptual changes. In such cases, the onset of motor incoordination with loss of short‐term memory and lengthening of reaction time is often observed. In addition, in some cases, some unpleasant effects are reported by users, such as panic attacks, anxiety crises, or rarely, hallucinations [8].

Although cannabis appears to possess beneficial effects in the human body, such as in the case of nausea following chemotherapy treatment, it can cause several adverse effects [9]. Regarding the documented acute effects associated with cannabis intoxication, there is wide variability in the literature depending on the age of the person developing the symptomatology. Adults typically present with cardiovascular (at low dose, hypertension and increased heart rate, and at higher dose, hypotension and bradycardia, coronary artery spasm, or myocarditis), respiratory (increased or decreased respiratory rate, depression, and apnea), ocular (nystagmus and hyperemia), gastrointestinal (dryness and hunger), and neurological (incoordination and drowsiness) effects [8, 10]. Infants, on the other hand, primarily present with incoordination, sensory changes, and lethargy in cases of severe encephalopathy and coma unresponsive to treatment. Chronic alterations related to prolonged use of these substances are also contemplated in the literature, but these are indistinguishable from those commonly caused by cigarette smoking or some environmental pollutants [1, 5, 11]. Regular cannabis exposure in children and adolescents, since their brains are still maturing, can interfere with the formation and strengthening of neural connections, potentially having long‐lasting effects on cognitive abilities, such as memory, learning, and attention, that impair school achievement. It also implies an increased risk of behavioral disorders, such as anxiety, depression, and other psychiatric problems. It is also associated with neuroimaging findings such as higher gray matter densities in the left nucleus accumbens [12, 13]. Other effects correlated with prolonged use include abnormalities of alveolar macrophages with alterations in the immune response concerning both antimicrobial activity and antitumor action [1, 11, 14].

The effects of THC vary, in each case, depending on the mode of intake, either inhalation (smoking or vapor) or oral (ingestion), whereas other modalities are also described [15, 16, 17]. Inhalation intake has a bioavailability of 18%–50% with a *C*_Max_ of up to 300 ng/mL, depending on the cigarette potency [18, 19, 20]. Effects appear within minutes, reach the peak after 30–60 min, and disappear within 60–180 min [21]. When ingested orally, THC has a bioavailability of 6%–20% and a *C*_Max_ of < 10 ng/mL, achieved in a longer time than inhaled intake [22]. The half‐life for both modes is 1–2 days in acute users and 5–13 days in chronic users [21]. THC has a volume of distribution (*Vd*) of 9–11 L/kg with a plasma‐to‐whole blood ratio of about 2, in view of its low partition coefficient in erythrocytes [5, 21].

Regarding its distribution, it exhibits high accumulation in high perfusion tissues such as the brain and slow uptake in low perfusion tissues such as adipose tissue, from which it is released in the same manner. The active ingredient is metabolized in the liver by two processes: hydroxylation and oxidation. Following the former, 11‐hydroxy‐Δ^9^‐tetrahydrocannabinol (11‐OH‐THC) is produced, whereas following the latter, the resulting substance is tetrahydrocannabinol (THC‐COOH). As for elimination, 70% of THC is excreted in the urine (30%) and feces (40%) [5]. Given that a significant number of metabolites are eliminated through the intestines, entero‐hepatic recirculation may occur. This could explain the slow elimination and long half‐life of THC, which can be found in urine as THC for days [5].

In agreement with literature data, the risk of death due to direct cannabis intoxication appears negligible. However, there are some cases where its use is linked to the possibility of fatal events, such as in the case of developing cardiac complications or physical injuries procured in an altered conscious state [4]. The most relevant cases involve adults, rather than children, with preexisting conditions, especially heart disease.

The authors present here a fatal case of a toddler in which cannabis was prominently involved in the determinism of death. The absence of preexisting conditions alone capable of causing his death, the circumstantial data, the absence of other possible causes, and the amount of the substance detected in the victim's fluids support this statement.

## 2 Case Report

### 2.1 Forensic Inspection

An infant (aged under 3 years old) was taken by their parents to the nearest hospital due to the onset of alterations in walking, balance, and consciousness. Upon admission of the infant, at about 2 p.m., health care providers learned from the father that the child had allegedly ingested a substance having the color of cork and the consistency of soil. According to his account, such ingestion occurred that morning while the infant was playing at the playground located near their home. On that occasion, realizing that the infant had ingested it, he approached the child and, opening their mouth, noticed the presence of unidentified material with the characteristics described above. After manually removing it from the child's mouth, he returned home to prepare lunch. Once over, he noticed that the child appeared staggering, unbalanced, and absent. Promptly contacting his wife, they transported the child to the nearest emergency room. Despite all the therapies implemented by the medical staff, the condition was progressively worsening with the onset of bradypnea and then dyspnea requiring intubation, so fentanyl (20 mcg) and ketamine (30 mg) were administered. At 4 p.m. the same day, death was noted. During the search carried out by judicial police officers at the family's home, a modest amount of hashish was found, of which the father appeared to be a user. From the testimonial information gathered, the mother used, up to 10 years earlier, heroin by intravenous administration.

### 2.2 Histopathological Findings

Autopsy examination revealed multiorgan congestion in the encephalic (congestion with cerebral edema), pulmonary (congestion with pulmonary edema), hepatic (sinusoidal congestion and cytoplasmic vacuolization), and adrenal (medullary congestion) districts. No cardiac abnormalities were detected either microscopically or macroscopically. During the autopsy examination, hair from the nuchal region, central and peripheral blood, bile, urine, and gastric contents were taken for toxicological analysis. The collected fluids and tissues were stored in 10‐mL polyethylene tubes with sodium fluoride as a preservative, whereas the hair was placed in an aluminum foil. Samples of brain, liver, and lung were also taken during the autopsy to be stored in formalin.

## 3 Materials and Methods

The autopsy specimens were subjected to a laboratory post‐mortem toxicological screening battery. In addition to autopsy tissues, fluids sampled in the emergency room (peripheral blood) were also included in this analysis. Narcotic and psychotropic substances were screened in cadaveric urine by immunochemical technique. In addition, xenobiotic substances were searched in living blood, cadaveric blood, urine, and bile by ultra‐high pressure liquid chromatography–multiple mass spectrometry (UPLC–MS/MS) on a Xevo TQS Waters. With the same instrument, the hair sample was analyzed for qualitative–quantitative detection of xenobiotic substances, including THC. Analyses on keratin matrices were conducted after the hair washing solution tested negative. THC, 11‐OH THC, and THC‐COOH were quantified in fluids and tissues by using gas chromatography–mass spectrometry (GC–MS) on an Agilent 5973 instrument.

In addition, a genetic analysis was also conducted on the heart muscle of the deceased for the research of mutations eventually related to the development of sudden arrhythmic events. However, this test resulted negative.

### 3.1 Screening of Narcotic and Psychotropic Substances in Cadaveric Urine by Immunochemical Technique

An automatic Viva Tween apparatus from the Siemens company, Milan, Italy, with EMIT reagents for immunochemical assays was used for the following classes of substances: opiates, cocaine, cannabinoids, amphetamine, ecstasy, benzodiazepine, barbiturates, and methadone. The sample volume used was 0.250 mL.

### 3.2 General Unknown Analysis of Xenobiotics in Blood, Urine, and Bile by UPLC–MS/MS

One milliliter of blood, bile, and urine, after addition of deuterated internal standards of the major substances of abuse, was made basic with phosphate buffer and extracted in 4 mL of ethyl acetate. The extract was brought to dryness under nitrogen, and the residue dissolved with 100 μL of water/acetonitrile 90/10 0.1% formic acid. Aliquots of 5 μL of extracts were injected into a Waters liquid chromatograph coupled with the Waters Xevo TQS micro instrument. The column used was an Atlantis T3 of 150 × 2.1 mm. Elution was carried out at a gradient of 0.1% formic acid water and 0.1% formic acid acetonitrile, flowing at 300 μL/min. Ionization was carried out by electrospray in positive or negative ion mode. Acquisition was under MS/MS conditions with collision of the pseudo molecular ions [M + H]^+^ or [M − H]^−^. The procedure allows the detection of more than 180 substances, including narcotics, stimulants, sedative‐hypnotics, hallucinogens, antidepressants, antipsychotics, and neuroleptics, and also includes xenobiotics of natural and industrial origin, with a lowest limit of identification varying from 1 to 10 ng/mL in blood.

### 3.3 Quali‐Quantitative Analysis of Xenobiotics in Hair by UPLC–MS/MS

Hair samples, 3 cm long, were sectioned, decontaminated with successive aliquots of methanol and water. They were dried and comminuted in a ball mill. Extraction was performed with 500 μL of M3 solvent by Comedical, incubating for 1 h at 80°C. After centrifugation two times at 13000 rpm, the supernatant was diluted with water for HPLC and injected into Waters liquid chromatograph coupled with Waters Xevo TQS instrument. The column used was an Atlantis T3 of 150 × 2.1 mm. Elution was carried out at a gradient of 0.1% formic acid water and 0.1% formic acid acetonitrile, flowing at 300 μL/min. Ionization was carried out by electrospray in positive ion mode. Acquisition was under MS/MS conditions with collision of the pseudo molecular ions [M + H]^+^ or [M − H]^−^. A 7‐point calibration curve was set up in parallel by the addition of all analytes of interest in known amounts to negative samples for quantitative interpretation. The substances identifiable by this method belong to the following classes: opiates (morphine, heroin, codeine, acetylcodeine, dihydrocodeine, and 6‐monoacetylmorphine), opioids (hydromorphone, hydrocodone, oxycodone, oxymorphone, and naloxone), synthetic opioid substitutes (methadone and buprenorphine), cannabinoids (tetrahydrocannabinol, cannabidiol, and cannabinol), cocaine (cocaine, benzoylecgonine, cocaethylene, and norcocaine), amphetamines (MDA, MDMA, MDEA, BDB, MBDB, amphetamine, and methamphetamine), amphetamine analogs, mephedrone, methylenedioxypyrovalerone, hallucinogens (ketamine, phencyclidine, and LSD), benzodiazepines and analogs (zolpidem, zopiclone, and zaleplon), antidepressants, antipsychotics, anesthetics, neuroleptics, and hypnotic sedatives, as well as other substances of toxicological interest of natural, pharmaceutical, and industrial origin.

### 3.4 Quantitative Analysis of Cannabinoids and Metabolites in Blood, Urine, Bile, and Tissues (Brain, Liver, and Lungs) by GC–MS

THC‐D3 and THC‐COOH‐D3 internal standards are added to 2 mL of blood, urine, and bile or 2 g of homogenized tissue. Glucuronide hydrolysis was carried out with 200 μL of 10 M KOH at 60°C for 20 min; pH was adjusted with 270 μL of glacial acetic acid and 2 mL of 50‐mM phosphoric acid. Extraction was then carried out with 5 mL of 90:10 hexane/ethyl acetate and 0.4% glacial acetic acid, for 15 min. The extract was brought to dryness under nitrogen, and the residue was treated with 50 μL of MSTFA 1% TCMS for 30 min in an oven at 75°C. Two microliters of derivatized was injected into an Agilent 5973 GC–MS instrument with column: HP5‐MS, 30 m × 0.25 mm × 25 μm; carrier gas: helium 0.800 mL/min. Gradient temperature program from 50°C to 300°C, spitless injection; the electron ionization conditions were 70 ev; the acquisition was in selected ion monitoring (SIM) for ions at m/z 306,389,374 (THC D3, IS), 303,371,386 (THC), 374,462,477 (11‐OH‐THC D3, IS), 371,459,474 (11‐OH‐THC), 476,374,491 (THC‐COOH D3 IS), and 303, 371,488 (THC‐COOH).

A 5‐point calibration curve was set up in parallel by addition of standards of THC, 11‐OH‐THC, and THC‐COOH in known amounts to blank samples, useful for quantitative interpretation.

### 3.5 Analysis of the Substance Confiscated at the Scene by the Police

The substance of resinous consistency collected from the victim's home turned out to be hashish with the active ingredient delta‐9‐THC at 28%.

### 3.6 Analysis of the Gastric Content by UPLC–MS/MS

An amount of 2.5 g of gastric content was homogenized. The supernatant was centrifuged and extracted with hexane/ethylacetate 90:10 for 15 min. The extract was dried and added to 100 μL of methanol. It was finally analyzed using the UPLC–MS/MS technique.

## 4 Results

From a qualitative point of view, the systematic toxicological investigation carried out following the previously described procedures detected the presence of cannabinoids, ketamine, norketamine, and fentanyl in the blood sampled in the emergency room. The presence of cannabinoids was also detected in central blood, peripheral blood, urine, bile samples, and organs (liver, brain, and lungs). Analysis performed on keratin matrices found the presence of tetrahydrocannabinol, cannabidiol, cannabinol, methadone and EDDP (2‐ethyllidene‐1,5 dimethyl‐3,3‐diphenyl pyrrolidine), cocaine and metabolites (benzoylecgonine and norcocaine), ketamine, norketamine, morphine, hydromorphone, 6‐monoacetylmorphine (6‐MAM), and fentanyl. Finally, a not negligible quantity of hashish emerged from the analysis of the gastric contents. The identified substances were then quantitatively determined either in blood collected in the emergency room and in cadaveric blood, bile, urine, and organs. The results are respectively listed in Tables 1 and 2. As for the hair, many substances were detected as detailed in Table 3.

**TABLE 1:** Results of analysis performed on blood collected in the emergency room.

Substance	Concentration (ng/mL)
Tetrahydrocannabinol (THC)	14
11‐Hydroxy‐tetrahydrocannabinol (11‐OH‐THC)	6.9
THC‐carboxylic acid (THC‐COOH)	77
Ketamine	726
Norketamine	265
Fentanyl	1.8

**TABLE 2:** Results of analysis performed on fluids and tissues sampled during the autopsy.

Specimen	THC	11‐OH‐THC	THC‐COOH
Peripheral blood	13.8 ng/mL	11 ng/mL	106 ng/mL
Central blood	3.6 ng/mL	3.8 ng/mL	40.0 ng/mL
Urine	4.6 ng/mL	3.8 ng/mL	51 ng/mL
Bile	Not estimable	60.8 ng/mL	247.6 ng/mL
Brain	19 ng/mL	Absent	1.2 ng/mL
Liver	Absent	9.8 ng/mL	81.0 ng/mL
Lung	Absent	Absent	40.0 ng/mL

**TABLE 3:** Results of hair analysis, 0‐ to 3‐cm segment.

Substance	Concentrations (pg/mg)
THC	910
Cannabidiol	30
Cannabinol	30
Methadone	25
EDDP	4
Cocaine	400
Benzoylecgonine	700
Norcocaine	4
Morphine	90
6‐Monoacetylmorphine	220
Hydromorphone	15
Ketamine	1.38
Norketamine	0.28
Fentanyl	0.001

Lastly, the gastric content amounted to 32 g as shown in Table 4. Of this amount, the grams of THC were calculated to be 290 mg. As the substance of resinous consistency collected from the victim's home turned out to be hashish with the active ingredient delta‐9‐THC at 28%, the total amount of hashish remaining in the stomach was estimated to be 1 g.

**TABLE 4:** Results of analysis of gastric content.

Sample	Total amount (g)	THC calculated (mg)	Total amount of hashish estimated in the stomach (g)
Gastric content	32	290	1

## 5 Discussion

Cannabis is the most widely used illegal drug but is rarely considered a causal factor in death. Indeed, its use‐related toxicity is considered insignificant in fatal cases [4]. In support of this theory, it should be considered that the psychotropic effects that occur when taken in high doses are quite unpleasant and, before dangerous reactions occur, tend to self‐limit further intake [23, 24]. For the above reasons, cases of adult individuals dying after cannabis assumption, regardless of the modality, are very rare, if not extremely rare. The only detectable cases in the literature involve adult subjects, often suffering from preexisting disease patterns. Drug‐induced deaths are rarely considered in the analysis of fatal cases involving children, even though the increasing legalization of drugs is leading to an increasing amount of drug intoxication even in young subjects.

Regarding the presence of THC and its metabolites 11‐OH‐THC and THC‐COOH in the blood sampled in the emergency room, the concentrations of 14, 6.9, and 77 ng/mL, respectively, are widely within the ranges reported in the literature as associated with cases of intoxicated subjects after cannabinoid intake [2, 25, 26]. It must be considered that the values obtained from toxicological analysis refer to whole blood and not plasma, as occurs in the relevant literature. Considering the low partition coefficient of erythrocytes [22], the concentrations measured in plasma are twice as high as those obtained in blood, and therefore, the latter should be considered doubled in view of performing an adequate comparison.

Furthermore, regarding the interpretive blood cut‐offs in use in major European countries for the assessment of disabling effects related to cannabinoid use in adults (Belgium–THC: 1 ng/mL; Luxembourg–THC: 1 ng/mL; Germany–THC: 1 ng/mL; United Kingdom–THC: 2 ng/mL) [27], it is evident that compared with these interpretive values, those found in our case are much higher.

Supporting the above is the finding of a symptomatology consistent with that known to be associated with intoxication by THC and the active metabolite 11‐OH‐THC [2]. In fact, from 1 p.m. onward, the child staggered with loss of balance and appeared absent. While being transported to the hospital, the child was inclined to fall asleep and, when admitted to the hospital, showed a tendency to doze off showing bradypnea with apnea.

Regarding the pharmacokinetic profile of cannabis, it is necessary to consider that the lower amount of adipose tissue typically present in children compared with adults justifies a lower distribution of the drug in that tissue resulting in higher concentrations in the blood than in adults, considering the same amount of absorbed substance [28]. In addition, children up to the age of 3 years possess a lower gastric pH than that of the adult, implying a higher bioavailability of THC due to less degradation of the substance at the gastric level [29].

According to a model proposed by Huestis in the living subject, it would be possible to cautiously estimate, based on the blood concentration of THC and THC‐COOH, the elapsed time since the last intake. In this case is estimated that the intake had occurred approximately 1:50 h (1 h 46′) before blood sampling. This result should be considered purely as indicative, since the formula was originally intended for estimating the elapsed time since intake in the case of adult subjects taking THC as a smoke. Moreover, Huestis' predictive model should be interpreted cautiously when applied to cadaveric samples, since THC is subject to post‐mortem redistribution.

Considering that cadaveric blood in forensic cases is not usually immediately sampled and the corpse is not immediately put in a rephrigerated environment, given both the redistribution phenomena and the degradative kinetics of THC in cadaveric blood [30], it can be hypothesized that blood THC concentration in the child moments before death was higher than that observed in the cadaveric sample.

Considering all of the above, it is believed that the toddler was in a state of acute cannabinoid intoxication at the time of the access to the emergency room.

In deaths studied in animal models with acute administration, the dose of THC that kills 50% of animals (LD50) is 40 mg/kg intravenously in the rat and 130 mg/kg in the dog and monkey [10]. Extrapolating from animal to human, it would result in an e.v. lethal dose of between 2.6 and 8.45 g THC for a 65‐kg adult. When administered orally, LD50 in rats is over 500 mg/kg, which is a human equivalent oral dose of about 806.45 mg/kg [31]. Considering a 10‐kg child, the lethal oral dose of THC would be approximately 8 g. In our case, 1 g of hashish (presumably at 28% THC) was measured in the gastric content. It is to be noted that the child ingested the substance a couple of hours before the admission to the hospital, so its enteric absorption had already begun, even if it was not complete at the time of death. It is reasonable to assume that the dose ingested was significantly higher, even if it is difficult to estimate.

Therefore, in the absence of other causes potentially responsible for death, it can be stated that the infant's death is causally attributable to the ingestion of cannabis.

Finally, according to fentanyl and ketamine pharmacokinetics [32, 33], the detection of fentanyl, ketamine, and norketamine at concentrations of 1.8, 726, and 265 ng/mL, respectively, is consistent with the documented administration of these sedatives at the dosage of 20 mcg (fentanyl) and 30 mg (ketamine) for orotracheal intubation maneuvers.

Regarding the autopsy sampling, the THC concentrations detected in the peripheral blood sample (6.9 ng/mL), considering the plasma‐to‐blood ratio as explicated in the previous paragraph, are well above those reported in literature as causative of death in adults without pathological preexisting conditions [3]. Furthermore, the urinary presence of THC [34, 35] and the metabolites 11‐OH‐THC and THC‐COOH at the concentrations of 4.6, 3.8, and 51 ng/mL, respectively, is indicative of a recent use of cannabis, as explained hereafter. Given the relatively high concentrations of THC and metabolites (THC‐COOH and 11‐OH‐THC) found in blood, the relatively low concentrations detected at the urinary level can be attributed to the initial and thus incomplete metabolism of the active ingredient THC and to the early stages of its excretion when death occurred [5], nor is any data available on the metabolic efficiency of the child.

As to keratin matrices, the detection of cannabinoids in head hair (THC, 0.91 ng/mg; CBD, 0.030 ng/mg; and CBN, 0.030 ng/mg) implies several mechanisms of incorporation: by blood capillaries, through sebum or sweat [22] or through hair contact with the sweat of a third active user [36]. Given the lipophilicity of the THC molecule, its ability to bind to hair melanin is reduced; the concentrations detected in hair are therefore lower than they would have been in the case of other substances of abuse [22]. The presence of fentanyl at a concentration of 0.001 ng/mg does not contrast with the documented administration of this sedative drug at a dosage of 20 mcg in the ER at about 2:10 p.m. As regards the detection of ketamine and its metabolite norketamine, in concentrations of 1.38 and 0.28 ng/mg, respectively, it is indicative of systemic exposure to the substance. On this point, it should be noted that the first‐aid report shows the administration of ketamine at a dose of 30 mg, necessary for the orotracheal intubation maneuvers. Given the high concentrations found, it is reasonable to infer that the incorporation of both THC and ketamine in the hair was due to the sweat produced by the subject in the agonic or preagonic phase. However, especially for cannabinoids, it cannot be excluded that there was also an exposure in the 3 months before death. Before considering the other drugs found in the hair of the victim, it should be noted that children's hair is finer and more porous than those of an adult, making them at greater risk of sweat contamination [37].

As regards cocaine, the absence of cocaine and its metabolites in the blood, urine, and bile reveals that the child was not exposed to cocaine recently and was not intoxicated by cocaine just before death. The same consideration holds for methadone, EDDP, morphine, codeine, and 6‐AM absent in the cadaveric fluids/tissues and present only in hair, thus excluding a recent acute heroin and methadone intoxication. However, the presence of metabolites (benzoylecgonine, norcocaine, EDDP, morphine, codeine, and 6‐AM) may suggest that the parent compounds underwent metabolism in the child's body prior to incorporation into hair. It is not possible, however, to determine whether these were taken by the young victim (e.g., by accidental ingestion) or by adults with whom the child had contact, resulting in the transfer from their sweat to the toddler's hair. However, based on the concentrations found on keratin matrices, it can be affirmed that the child had certainly been exposed to heroin, cocaine, and methadone either actively or passively.

Considering THC concentrations in fluids, these are compatible with the development of symptoms of acute central neuronal intoxication. In fact, from early afternoon, the child presented recurrent episodes of bradypnea with subsequent apnea. The acute toxicity of cannabis is due to THC and is mediated by its effects on the neuronal system. In fatal cases caused by acute administration and derived from animal models, apnea always occurs or cardiac arrest if apnea is prevented [10]. Data from the analysis of the liver sample, which resulted in the detection of 11‐OH‐THC and THC‐COOH at concentrations of 9.8 and 81.0 ng/mg, respectively, indicate that cannabis has been ingested, absorbed, and metabolized in the liver.

## 6 Conclusions

The increasing legalization of cannabis worldwide is leading to a growing number of problems, including a surge of intoxication cases. Analysis of data available in the literature shows that, in most cases, the intoxicated are adults with preexisting medical conditions, whereas it is rare to find lethal cases in adults or children. Since few other case reports in the literature have addressed the analysis of children who died due to cannabis use, this article proposes an investigation of a particular case of death certainly attributable to the ingestion of considerable doses of cannabis derivative (hashish).

In the case presented, the toxicological analysis performed on the child's biological fluids (blood, urine, and bile) allowed us to conclude that at the time of admission, the child was certainly in a state of acute intoxication by cannabis. Given the concentration of THC found in the blood collected both in the emergency room and during the autopsy, considering those reported in literature as “lethal,” death is believed to be causally attributable to cannabis ingestion.

The data from hair analyses allowed us to state that the child had already been exposed to multiple substances of abuse, reasonably used by their parents: cannabis, heroin, cocaine, and methadone.

In conclusion, although further studies are needed to better elucidate the specific role of cannabis in the determinism of death, since for this substance no pathognomonic effects are produced in the body, the premortal and postmortal investigations are consistent with the hypothesis of a fatal cannabis ingestion.

## Conflicts of Interest

The authors declare no conflicts of interest.

## Data Availability

The data that support the findings of this study are available on request from the corresponding author. The data are not publicly available due to privacy or ethical restrictions.
